# Prognostic impacts of soluble immune checkpoint regulators and cytokines in patients with SARS-CoV-2 infection

**DOI:** 10.3389/fimmu.2022.903419

**Published:** 2022-08-15

**Authors:** Nuri Lee, Seri Jeong, Kibum Jeon, Min-Jeong Park, Wonkeun Song

**Affiliations:** ^1^ Department of Laboratory Medicine, Hallym University College of Medicine, Seoul, South Korea; ^2^ Department of Laboratory Medicine, Hallym University College of Medicine, Hangang Sacred Heart Hospital, Seoul, South Korea

**Keywords:** immune checkpoint, COVID-19, cytokine release syndrome, SARS-CoV-2, prognosis

## Abstract

Coronavirus disease 2019 (COVID-19) has been a pandemic for the past two years. Predicting patient prognosis is critical. Although immune checkpoints (ICs) were shown to be involved in severe acute respiratory syndrome coronavirus 2 (SARS-CoV-2) infection, quantitative studies of ICs in clinical practice are limited. In this study, various soluble ICs (sICs) and cytokine levels in patients with SARS-CoV-2 infection at different time points were compared between survivors and deaths; we also examined whether sICs are useful for predicting prognosis. sICs and cytokines were measured in serum samples from 38 patients diagnosed with COVID-19 in the first and second week post-diagnosis. All assays were performed by bead-based multiplexed immunoassay system using Luminex Bio-Plex 200 system. The correlation of sICs and cytokines with laboratory markers was evaluated, and the levels of sICs in survivors were compared with those in deaths. Among the sICs, the second-week levels of soluble cluster of differentiation (sCD27, p = 0.012), sCD40 (p< 0.001), cytotoxic T-lymphocyte-associated protein 4 (sCTLA-4, p< 0.001), herpes virus entry mediator (sHVEM, p = 0.026), and T-cell immunoglobulin and mucin-domain containing-3 (sTIM-3, p = 0.002) were significantly higher in deaths than in survivors. The levels of nine cytokines assessed in the second week of deaths were significantly higher than those in survivors. The sICs sCD27, sCD40, sCTLA-4, and sTIM-3 and cytokines chemokine CC motif ligand 2 (CCL2), GM-CSF, IL-10, and IL-8 showed significant positive correlations with the levels of C-reactive protein (CRP) and procalcitonin and were negatively correlated with the absolute lymphocyte count and platelet values. Increased levels of sICs including sCD27, sCD40, sCTLA-4, and sTIM-3 and cytokines were significant factors for poor prognosis. sICs, together with cytokines and inflammatory markers, may be useful as prognostic stratification markers in SARS-CoV-2-infected patients.

## Introduction

The coronavirus disease 2019 (COVID-19) pandemic has continued for more than two years, highlighting the importance of disease diagnosis, evaluation of the patient’s status and prognosis, screening for severely ill patients, and implementing effective treatment methods. In this context, immune checkpoint (IC) molecules have emerged as important in the mechanism of severe acute respiratory syndrome coronavirus 2 (SARS-CoV-2) infection and its progression. Several review articles reported the potential of using ICs as biomarkers of viral infections, including SARS-COV-2 infection (1, 2). ICs are co-stimulatory and co-inhibitory signaling molecules expressed on immune cells and are involved in regulating T-cell activation. Once triggered, these molecules function in either the potent immune response by releasing proinflammatory mediators or by breaking the immune system, maintaining self-tolerance, and preventing immunopathology in the body. In patients with cancer or viral infections, the IC pathway causes T-cell dysfunction to affect immune escape (2–6). Additionally, IC-targeted treatments have been suggested as alternatives for SARS-CoV-2 infection (7–9).

Although ICs were shown to play important roles in SARS-CoV-2 infection, few clinical studies have focused on quantitatively evaluating ICs in patients with SARS-CoV-2 infection. Additionally, the pattern of changes in IC values with disease progression and their prognostic significance remain unclear. Measurement of soluble ICs (sICs) and/or staining of tissue samples revealed a moderate to severe association between increased levels of ICs and disease severity in patients with COVID-19. However, few sICs, such as programmed cell death protein 1 (PD-L1), were measured, and overall survival (OS) was not analyzed because patients who died were excluded from the study (10, 11). Moreover, the sequential changes in sICs during hospitalization are not well-understood (10), and the time point at which sICs were measured was ambiguous in most studies (11). The associations of sICs with common inflammatory markers and cytokines are unclear. Therefore, studies are needed to measure various ICs at different time points in patients with COVID-19.

This study was conducted to determine the importance of sICs as prognostic predictors in patients with SARS-COV-2 infection. The levels of sICs and cytokines in patients with SARS-CoV-2 infection were compared between survivors and deaths. In addition, the levels of sICs and cytokines in each patient were measured in the first and second weeks post-diagnosis to investigate their changes over time. Furthermore, different sICs were evaluated at varying time points to identify the ideal measurement time for determining the patient’s status and disease prognosis. Finally, the cytokines values and laboratory test results were obtained at the time of sIC measurements, and the relationship and prognostic effect of these markers were investigated through an integrated analysis of various prognostic factors.

## Materials and methods

### Study population

From September 2020 to July 2021, 38 patients diagnosed with COVID-19 and treated in the intensive care unit of Kangnam Sacred Heart Hospital were enrolled in the study. The inclusion criteria for this study were as follows: age > 20 years, confirmed for SARS-COV-2 infection, treated in the intensive care unit (ICU). Patients with insufficient quantities of residual serum samples were excluded. COVID-19 was diagnosed in patients with SARS-CoV-2 RNA using real-time reverse transcription-polymerase chain reaction from nasopharyngeal and/or throat swab samples. Residual serum samples were collected from these patients during the first and second weeks after the diagnosis of COVID-19. A total of 76 specimens were collected from 38 patients. An aliquot (1.0 mL) of each sample was transferred into microtubes and stored at -70°C prior to measuring the concentration of sICs and cytokines. The study was approved by the Institutional Review Board of Kangnam Sacred Heart Hospital of Hallym University, Seoul, Korea (No. 2020-08-004-003). The requirement for informed consent for this study was waived by the institutional review board because the anonymity of personal information was maintained.

### Quantification of sIC regulators and cytokines

sICs in the serum were quantified using Milliplex Human Immuno-Oncology Checkpoint Protein Premixed 17-plex Panel 1 (Millipore, Billerica, MA, USA). The sIC panel included soluble cluster of differentiation 27 (sCD27), sCD40, sCD80/B7-1, sCD86/B7-1, soluble B-lymphocyte and T-lymphocyte attenuator (sICOS), soluble cytotoxic T-lymphocyte-associated protein 4 (sCTLA-4), sBTLA, soluble B-lymphocyte and T-lymphocyte attenuator (sBTLA), soluble herpes virus entry mediator (sHVEM), soluble programmed cell death protein 1 (sPD-1), soluble programmed death-ligand 1 (sPD-L1), sPD-L2, sPD-L3, soluble glucocorticoid-induced TNFR-related protein (sGITR), soluble ligand for receptor TNFRSF18/AITR/GITR (sGITRL), soluble Toll-like receptor 2 (sTLR-2), soluble T-cell immunoglobulin and mucin-domain containing-3 (sTIM-3), and lymphocyte activation gene 3 (sLAG-3). Cytokines levels were measured using a Human XL Cytokine Luminex Performance Panel Premixed Kit (R&D Systems, Minneapolis, MN, USA). The cytokine panel included chemokine CC motif ligand 2 (CCL2), CCL3, CCL4, C-X-C motif chemokine ligand (CXCL10), granulocyte-macrophage colony-stimulating factor (GM-CSF), interferon (IFN)-α, IFN-γ, interleukin (IL)-10, IL-12p70, IL-13, IL-17A, IL-1α, IL-1β, IL-4, IL-6, IL-8, and tumor necrosis factor-α (TNF-α). All assays were conducted using Luminex-based multiplex technology according to the manufacturer’s protocols on a Luminex 200 Bio-Plex instrument (Bio-Rad, Hercules, CA, USA).

### Laboratory assessments

Clinical data were retrospectively investigated using medical records, and quantitative values were measured at the same time as sICs/cytokines estimation in the first and second weeks. Blood urine nitrogen (BUN), creatinine, lactate dehydrogenase (LD), procalcitonin, and C-reactive protein (CRP) levels were determined using an Atellica IM (Siemens Healthcare Diagnostics Manufacturing Ltd., Munich, Germany). The hemoglobin, platelet, total white blood cell (WBC), absolute neutrophil count (ANC), and lymphocyte counts were obtained using an ADVIA 2120i (Siemens Healthcare Diagnostics Manufacturing Ltd.). SARS-CoV-2 RNA was extracted using the MagNa Pure 96 System (Roche Diagnostics, Basel, Switzerland) and subjected to a real-time polymerase chain reaction using the STANDARD M nCoV Real-Time Detection kit (SD Biosensor, Gyeonggi, South Korea) and Bio–Rad CFX96 analyzer (Bio–Rad Laboratories) for quantification.

### Statistical analyses

The clinical characteristics, sICs levels, and cytokines levels of the enrolled patients were compared between survivors and deaths. Moreover, differences between the survivor and death groups were compared based on the values measured in the first and second weeks. Mann–Whitney U test was used to evaluate statistical significance. Pearson’s correlation coefficient was used to analyze the correlation between laboratory markers and various sIC and cytokines levels. The degree of correlation was considered weak for values of 0.10 ≤ r< 0.30, moderate for values of 0.30 ≤ r<0.50, and strong for values of r ≥ 0.50, as previously described (12). The Cox proportional hazards model was used to evaluate the prognostic impact of laboratory tests and sIC and cytokines levels on OS. Age, sex, ANC, and underlying diseases as factors that may influence prognosis were adjusted through multivariable Cox analysis (13). Cumulative OS curves for the cluster groups were calculated using the Kaplan–Meier method and compared using the log-rank test. The cutoff for continuous values was estimated using the Youden index. Pearson’s correlation coefficient and OS were analyzed using the values measured in the second week for all patients. Several factors affecting the survival rate of patients with SARS-CoV-2 infection were identified and classified using the K-means clustering method. Differences were considered statistically significant at p< 0.05. A corrected p-value using Bonferroni-adjustment was applied with the number of comparisons made (14). All statistical analyses were conducted using the statistical R-project program (version 3.6.2), PASW statistics version 18 (SPSS, Inc., Chicago, IL, USA), and MedCalc version 12.0 (MedCalc Software, Mariakerke, Belgium).

## Results

### Patient characteristics, sIC regulators, and cytokines in patients with COVID-19

Among the 38 patients with SARS-CoV-2 infection, 23 survivors and 15 deaths were included in the study. The median ages of the survivors and deaths were 67.0 and 76.0 years, respectively (p = 0.018). Among the survivors, creatinine (p< 0.001), CRP (p< 0.001), and sPD-L2 (p = 0.040) levels were significantly lower in the second week than in the first week. And, sCD80 (p = 0.029), sGITRL (p = 0.039), and sICOS (p = 0.018) levels were significantly higher in the second week than in the first week (**Table 1**). After adjusted by Bonferroni’s correction, creatinine (p = 0.019) and CRP (p = 0.005) remained for statistically significant factors. Among the deaths, the total WBC (p = 0.004), ANC (p = 0.002), and sCD40 (p = 0.024) levels were significantly higher in the second week than in the first week. The above items were not statistically significant after adjusted by Bonferroni’s correction. When comparing the survivors with deaths, WBC (p = 0.004), BUN (p = 0.008), LD (p< 0.001), and CRP (p< 0.001) levels measured in the second week were significantly higher in survivors than in those who died. Of the sICs, sCD27 (p = 0.012), sCD40 (p< 0.001), sCTLA-4 (p< 0.001), sHVEM (p = 0.026), and sTIM-3 (p = 0.002) assessed in the second week were significantly increased in deaths compared to in survivors. In contrast, sLAG-3 (p = 0.038) levels decreased in the deaths. After adjusted by Bonferroni’s correction, CRP (p = 0.004), sCD40 (p<0.001), and CTLA-4 (p = 0.024) showed stastistical significance (**Table 1**). The differences in sex, Ct values for SARS-CoV-2 real-time reverse transcription-polymerase chain reaction tests, lymphocyte count, hemoglobin, and procalcitonin levels between the survivors and deaths and/or first and second weeks were not significant (**Table 1**). The levels of nine cytokines (CCL2, CCL4, CXCL10, GM-CSF, IL-10, IL-17A, IL-6, IL-8, and TNF-α) in deaths were significantly increased in the second week compared to those in survivors. CCL2 (p< 0.001), CXCL10 (p = 0.002), GM-CSF (p = 0.008), IL-10 (p = 0.007), and IL-8 (p = 0.009) showed stastistical significance after adjusted with Bonferroni’s correction. Among the survivals, after adjusted by Bonferroni’s correction, CXCL10 (p< 0.001), GM-CSF (p = 0.002), and IL-10 (p = 0.012) levels were significantly decreased in the second week compared to those in the first week (**Table 2**).

**Table 1:** Characteristics and levels of soluble type immune checkpoint regulators in survivors and deaths with SARS-CoV-2 infection.

Variables	Survivors (N = 23)			Deaths (N = 15)			p-value^⧉^
	1^st^ week	2^nd^ week	p^‡^	1^st^ week	2^nd^ week	p^‡^	
Age, year	67.0 (51.0–74.8)			76.0 (70.5–78.8)			0.018
Gender (male: female)	17:6			8:7			0.197
Underlying diseases^*^ (HTN: DM: CV: TB: others: none)	11:5:0:0:3:11			4:5:1:1:2:6			0.6398
rRT-PCR, Ct	25.6 (17.8–30.5)	29.8 (25.0–31.3)	0.063	24.7 (18.4–30.3)	26.5 (23.3–31.9)	0.320	0.964/0.776
Hemoglobin (g/dL)	13.9 (12.5–14.9)	13.2 (10.7–14.1)	0.170	13.0 (11.4–13.7)	11.3 (9.9–13.1)	0.236	0.152/0.221
Total white blood cell count (×10^9^/L)	7.07 (5.11–9.49)	8.7 (5.91–14.21)	0.147	6.44 (3.41–11.26)	13.0 (10.8–18.3)	**0.112**	0.858/**0.420**
Absolute neutrophil count (×10^9^/L)	6.45 (3.94–9.06)	6.73 (5.1–12.59)	0.301	8.21 (3.31–10.19)	11.4 (10.1–16.3)	**0.056**	0.483/0.4142
Lymphocyte count (×10^9^/L)	0.66 (0.37–0.98)	0.85 (0.44–1.20)	0.240	0.47 (0.40–0.60)	0.61 (0.39–0.69)	0.619	0.317/0.060
Platelet (×10^9^/L)	187 (151.3–241.8)	264 (185–318)	0.053	149 (105.5–200.8)	110 (83.5–217.3)	0.309	0.100/**0.056**
Blood urine nitrogen (mg/dL)	16.9 (12.1–25.3)	17.1 (15.2–23.5)	0.429	20.6 (16.8–27.5)	26.1 (22.4–34.8)	0.056	0.165/**0.224**
Creatinine (µmol/L)	0.77 (0.66–0.98)	0.61 (0.54–0.68)	**0.019**	0.75 (0.65–0.84)	0.69 (0.51–0.81)	0.237	0.378/0.54
Lactate dehydrogenase (IU/L)	443 (286–499)	286 (216–349)	0.067	475 (378–577)	493 (383–731)	0.520	0.094**/0.02**
Procalcitonin (ng/mL)	0.09 (0.06–0.29)	0.13 (0.10–1.3)	0.357	0.15 (0.08–0.87)	0.48 (0.30–1.26)	0.192	0.483/0.414
CRP (mg/dL)	119.8 (46.9–201.6)	16.3 (2.20–46.0)	**0.005**	91.8(53.3–132.9)	125.6 (55.7–224.7)	0.256	0.467**/0.004**
sBTLA (pg/mL)	23.0 (20.3–30.3)	27.0 (22.0–29.5)	0.373	21.0 (19.3–25.8)	30.0 (23.3–37.8)	0.053	0.401/0.275
sCD27 (pg/mL)	475 (361.6–1129.4)	556 (248–1205)	0.991	1046 (775.8–1344.9)	1553 (943–2613)	0.078	0.086/**0.336**
sCD28 (pg/mL)	44.0 (35.3–56.3)	47.0 (30.8–67.0)	0.818	52.0 (34.0–64.0)	58.0 (51.0–66.6)	0.221	0.687/0.083
sCD40 (pg/mL)	1301 (1116–1562)	1377 (1105–1689)	0.856	1927 (1693–2454)	2770 (2308–3308)	**0.672**	**0.026/<0.001**
sCD80/B7-1 (pg/mL)	46.0 (38.3–61.3)	71.0 (42.0–107)	**0.812**	54.0 (40.5–75.0)	47.0 (36.3–64.9)	0.480	0.324/0.165
sCD86/B7-2 (pg/mL)	24.0 (21.0–30.0)	29.0 (21.3–35.3)	0.153	24.0 (22.1–28.8)	24.0 (19.3–39.9)	0.836	0.653/0.323
sCTLA-4 (pg/mL)	50.0 (49.0–53.8)	54.0 (45.0–58.9)	0.560	60.0 (52.0–66.0)	65.0 (59.5–67.0)	0.191	**0.269/0.024**
sGITR (pg/mL)	105 (96.5–115.3)	101 (93.8–130.0)	0.939	133 (106.0–141.8)	131.5 (111–138.5)	1.000	**0.616**/0.064
sGITRL (pg/mL)	34.0 (29.1–44.1)	49.0 (28.8–60.3)	**1.000**	39.0 (27.3–48.0)	39.5 (23.0–54.5)	0.950	0.611/0.262
sHVEM (pg/mL)	8008 (6765–9081)	7778 (6022–8386)	0.606	8411 (7084–9655)	9600 (8438–10,021)	0.330	0.276/**0.728**
sICOS (pg/mL)	30.0 (25.0–28.0)	41.0 (32.3–70.9)	**0.504**	36.0 (27.0–49.5)	34.0 (26.3–56.3)	0.983	0.464/0.135
sLAG-3 (pg/mL)	970 (857–1505)	1558 (891–2241)	0.121	1073 (393–1995)	673 (271–1727)	0.494	0.870/**1.000**
sPD-1 (pg/mL)	71.0 (57.9–89.5)	80.0 (61.5–97.8)	0.398	72.0 (64.3–80.3)	83.0 (59.8–106.5)	0.254	0.811/0.622
sPD-L1 (pg/mL)	51.0 (40.3–60.0)	52.0 (40.1–71.8)	0.733	53.0 (48.0–69.0)	58.0 (41.5–72.1)	0.917	0.473/0.800
sPD-L2 (pg/mL)	6875 (5644–7539)	6221 (4629–6524)	**1.000**	6933 (5714–7430)	6269 (5892–6639)	0.272	0.917/0.420
sTIM-3 (pg/mL)	3472 (2478–4134)	3076 (1869–4079)	0.328	4001 (3547–5073)	5369 (4494–5808)	0.065	**1.000**/**0.056**
sTLR-2 (pg/mL)	157 (126.3–187.0)	166 (112.1–223.8)	0.475	165 (144.8–200.0)	231 (145.8–262.3)	0.093	0.296/0.066

Values are presented as median (interquartile range)

^⧉^The p-values before and after the slash (/) indicate the significance of the difference between survival and death in the first and second week, respectively.

^‡^p-value indicates the significance of the difference between the first and second weeks.

^*^Underlying diseases included hypertension (HTN), diabetes (DM), cardiovascular diseases (CV), tuberculosis (TB), and others (hyperlipidemia, hypothyroidism, and asthma). Patients with two or more diseases in one patient were included.

The p-values after adjusted by Bonferroni correction presented as bold letters.

CD, cluster of differentiation; CTLA-4, cytotoxic T-lymphocyte-associated protein 4; GITR, glucocorticoid-induced TNFR-related protein; GITRL, ligand for receptor TNFRSF18/AITR/GITR; HVEM, herpes virus entry mediator; ICOS, inducible T-cell co-stimulator; LAG-3, lymphocyte-activation gene 3; PD-1, programmed cell death protein 1; PD-L1, programmed death-ligand 1; rRT-PCR, real-time reverse transcription-polymerase chain reaction; sBTLA, soluble B-lymphocyte and T-lymphocyte attenuator; sICOS, soluble inducible T-cell co-stimulator; sPD-1, soluble programmed cell death protein 1; TIM-3, T-cell immunoglobulin and mucin-domain containing-3; TLR-2, Toll-like receptor 2.

**Table 2:** Levels of cytokines in patients with survivors and deaths of SARS-CoV-2 infection.

Variables	N^*^	Survivors (N = 23)			Deaths (N = 15)			p-value^⧉^
		1^st^ week	2^nd^ week	p^‡^	1^st^ week	2^nd^ week	p^‡^	
CCL2 (pg/mL)	38	385.4 (220.4–600.8)	231.5 (205.5–335.8)	0.106	509.4 (360.1-934.8)	794.4 (531.5–1306.7)	0.141	0.051/<**0.001**
CCL3 (pg/mL)	8	N/A			N/A			N/A
CCL4 (pg/mL)	37	168.3 (128.2–217.1)	128.2 (103.9–168.3)	0.053	185.7 (133.5–228.0)	177.1 (149.3–228.0)	0.901	0.565/**0.085**
CXCL10 (pg/mL)	38	716.4 (377.8–1422.2)	74.0 (52.9–233.3)	**<0.001**	843.1 (328.5–1607.0)	471.5 (299.4–635.4)	0.254	0.643/**0.002**
GM-CSF (pg/mL)	32	117.1 (71.7–181.8)	16.5 (1.93–58.0)	**0.002**	124.9 (69.9–194.8)	97.1 (60.8–149.2)	0.320	0.570/**0.008**
IFN-α (pg/mL)	8	N/A			13.9 (1.49–38.5)	0.92 (0.34–5.14)	**0.731**	N/A
IFN-γ (pg/mL)	0	N/A			N/A			N/A
IL-10 (pg/mL)	32	105.8 (66.3–203.5)	53.0 (8.88–66.3)	**0.012**	186.4 (92.7–280.6)	117.4 (75.2–236.1)	0.547	0.131/**0.007**
IL-12p70 (pg/mL)	2	N/A			N/A			N/A
IL-13 (pg/mL)	15	37.2 (21.0–43.5)	29.9 (21.0–40.2)	0.836	7.07 (7.07–17.6)	21.0 (7.07–29.9)	0.231	0.099/0.203
IL-17A (pg/mL)	27	1.95 (1.15–2.76)	0.33 (0.33–1.95)	0.082	1.95 (1.15–3.36)	1.95 (1.95–3.16)	0.530	0.940/**0.765**
IL-1α (pg/mL)	23	5.74 (4.03–11.6)	2.93 (1.22–5.70)	0.054	4.39 (1.65–6.69)	5.74 (2.93–6.69)	0.617	0.188/0.301
IL-1β (pg/mL)	14	N/A			2.13 (0.72–4.03)	2.43 (2.43–2.73)	0.668	N/A
IL-4 (pg/mL)	0	N/A			N/A			N/A
IL-6 (pg/mL)	26	37.7 (14.2–118.8)	9.23 (5.71–74.5)	0.112	46.9 (20.1–113.6)	75.6 (49.8–160.4)	0.141	0.665/**0.204**
IL-8 (pg/mL)	38	18.5 (13.3–39.6)	15.6 (10.3–34.3)	0.560	25.3 (19.0–48.9)	46.7 (28.0–77.8)	**0.612**	0.110**/0.009**
TNF-α (pg/mL)	31	7.83 (4.9–12.8)	5.39 (2.02–11.8)	0.193	7.83 (3.69–15.0)	11.79 (8.58–16.5)	0.237	0.904/**0.697**

Values are presented as the median (interquartile range).

^*^Total number of patients available for measuring cytokine levels.

^⧉^The p-values before and after the slash (/) indicate the significance of the difference between survival and death in the first and second week, respectively.

^‡^p-value indicates significance of the difference between the first and second weeks.

The p-values after adjusted by Bonferroni correction presented as bold letters.

CCL, chemokine CC motif ligand; CXCL, C-X-C motif chemokine ligand; GM-CSF, granulocyte-macrophage colony-stimulating factor; IFN, interferon; IL, interleukin; TNF, tumor necrosis factor.

### Correlation of laboratory test markers with cytokines and sIC regulators

**Figure 1:**
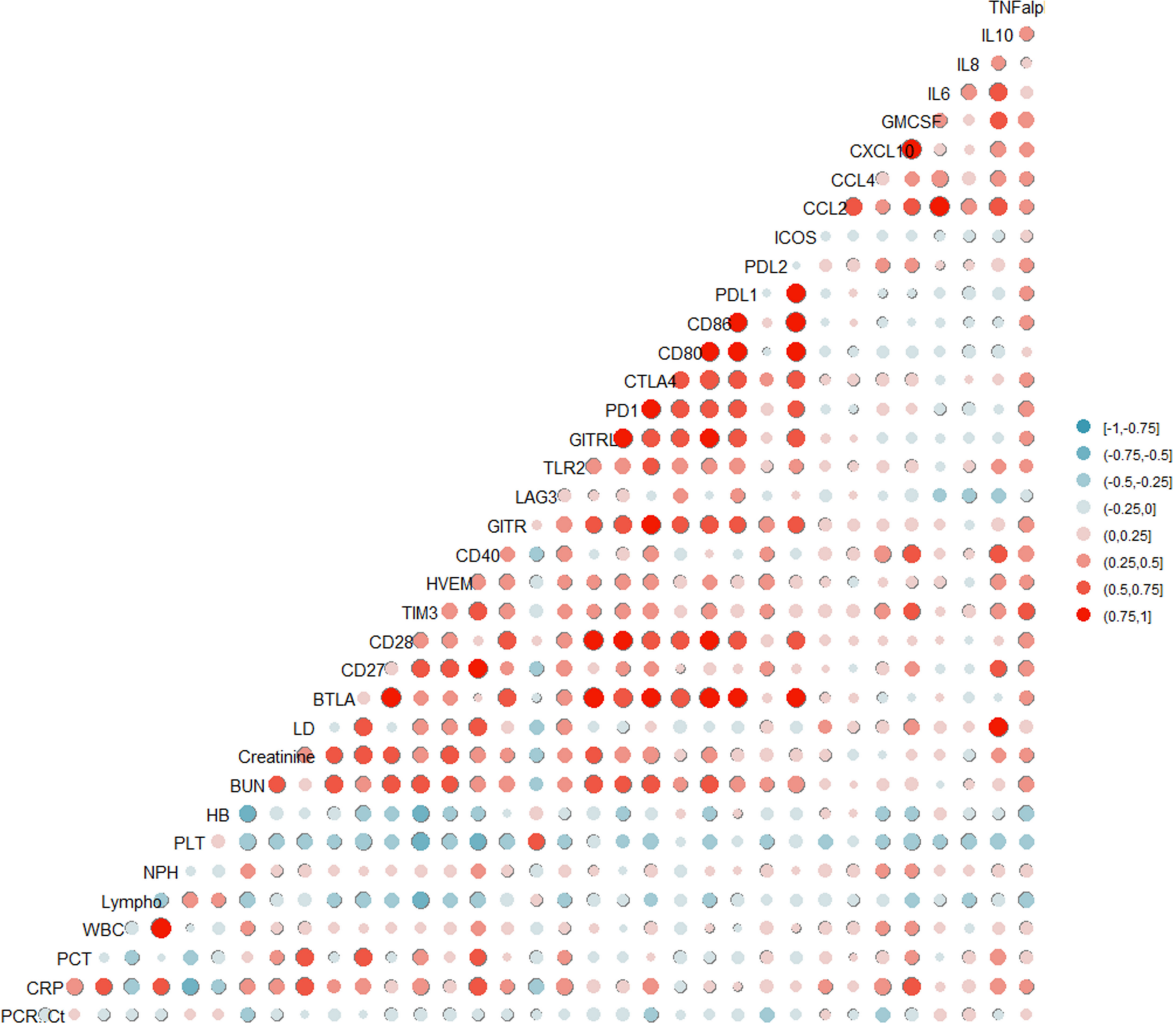
Pairwise association between clinical laboratory markers with soluble type immune checkpoint (IC) regulators and cytokines.

Inflammatory markers in patients infected with SARS-CoV-2 were positively correlated with the levels of some sICs and cytokines. The levels of sCD27 (r = 0.452, p = 0.006), sCD40 (r = 0.649, p< 0.001), sCTLA-4 (r = 0.452, p = 0.006), sTIM-3 (r = 0.498, p = 0.002), soluble Toll-like receptor-2 (r = 0.468, p = 0.004), CXCL10 (r = 0.495, p = 0.002), GM-CSF (r = 0.544, p = 0.0019), and IL-10 (r = 0.395, p = 0.031) were positively correlated with CRP levels (**Figure 1**). In contrast, LAG-3 levels were negatively correlated with CRP levels (r = -0.387, p = 0.020). In addition, sCD27 (r = 0.762, p = 0.006) and sCD40 (r = 0.643, p = 0.033) levels were positively correlated with procalcitonin. Fourteen sICs and 2 cytokines were positively correlated with the BUN and/or creatinine levels. Analysis of the CBC showed that sICs, including sCD27 (r = -0.416, p = 0.009), sCD28 (r = -0.352, p = 0.03), sCD40 (r = -0.367, p = 0.023), sHVEM (r = -0.345, p = 0.034), and sTIM-3 (r = -0.572, p< 0.001), were negatively correlated with the lymphocyte count. In addition, sCD27 (r = -0.471, p = 0.003), sCD28 (r = -0.327, p = 0.045), sCD40 (r = -0.511, p = 0.001), sHVEM (r = -0.392, p = 0.0154), sTIM-3 (r = -0.655, p< 0.001), CCL2 (r = -0.327, p = 0.045), CTLA4 (r = -0.457, p = 0.004), GM-CSF (r = -0.451, p = 0.01), IL-10 (r = -0.444, p = 0.011), and IL-8 (r = -0.399, p = 0.013) were negatively correlated with the platelet count. sCD40 was positively correlated with the total WBC count (r = 0.341, p = 0.036) and ANC (r = 0.346, p = 0.034). sLAG-3 expression was positively correlated with the platelet count (r = 0.584, p< 0.001) (**Figure 1**).

### Prognostic impact of IC regulators and cytokines

**Figure 2:**
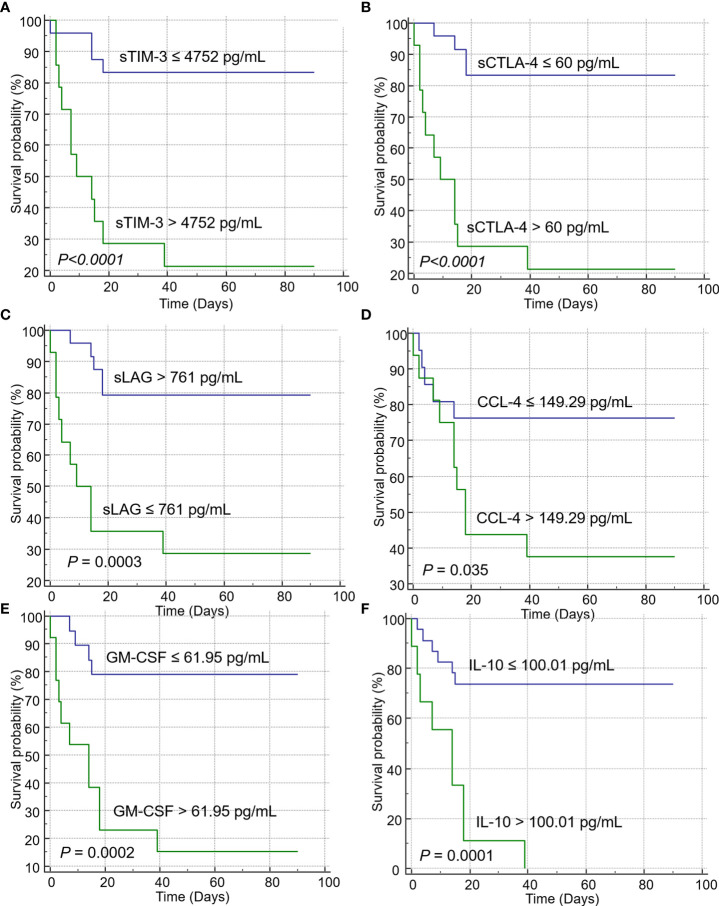
Kaplan–Meier curves showing the overall survival of patients with immune checkpoints (ICs) and cytokines. Increased levels of ICs such as **(A)** sTIM-3, and **(B)** sCTLA-4 were significantly associated with poor prognosis in the log-rank test. Decreased levels of **(C)** sLAG were correlated with poor prognosis. Increased levels of the cytokines **(D)** CCL-4, **(E)** GM-CSF, and **(F)** IL-10 were associated with poor prognosis.

Univariate analysis revealed that increased levels of sICs, including sCD27, sCD40, sTIM3, and sCTLA-4, and increased levels of cytokines, such as CCL2, CCL4, CXCL10, GM-CSF, IL-10, and IL-8, were associated with poor OS in patients with SARS-CoV-2 infection, whereas increased sLAG-3 levels were associated with a favorable prognosis. Increased age and total WBC, ANC, LD, and CRP levels were also associated with a poor prognosis. These sICs and cytokines were adjusted for age, sex, ANC, and comorbidities including hypertension, diabetes, cardiovascular disease, tuberculosis, and others (hyperlipidemia, hypothyroidism, and asthma) for multivariate analysis; all five sICs (sCD27, sCD40, sTIM3, sCTLA-4, and LAG-3) and four cytokines (CCL2, GM-CSF, IL-10, and IL-8) were significantly associated with OS. Increased LD and CRP levels were also associated with a poor OS in multivariate analysis (**Supplemental Table 1**). Using the Youden index, each factor was divided into two groups according to the prognosis. Increased level of sCD40 over 1804 pg/mL (Hazard ration (HR) = 6.36, p = 0.031), sCTLA-4 over 60 pg/mL (HR= 5.46, p=0.0097), sTIM-3 over 4752 pg/mL (HR= 5.13, p=0.014), CCL2 over 344.26 pg/mL (HR= 23.6, p=0.006), CXCL10 over 157.54 pg/mL (HR= 14.221, p=0.015), GM-CSF over 61.95 pg/mL (HR= 14.221, p=0.015), and IL-10 over 100.01 pg/mL (HR= 3.95, p=0.027) and decreased sLAG-3 not greater than 761 pg/mL (HR= 5.46, p=0.0097) were associated with poor OS (**Table 3**). Kaplan–Meier plots of patients with increased or decreased sICs and cytokine levels, classified using the Youden index, are shown in **Figure 2**. Patients with increased levels of sTIM3 (p< 0.0001), sCTLA4 (p< 0.0001), CCL4 (p = 0.035), GM-CSF (p = 0.0002), and IL-10 (p = 0.0001) exhibited a significantly poor prognosis, and those with decreased sLAG (p = 0.0003) levels showed a significantly poor prognosis. Additionally, increased levels of sCD27 (>355 pg/mL, p = 0.0113), sCD40 (>1804 pg/mL, p< 0.0001), CCL-2 (>344.26 pg/mL, p< 0.0001), and IL-8 (>21.86 pg/mL, p = 0.0005), with lesser than three survivors, were associated with poor prognosis.

**Table 3:** Cox proportional hazard model for factors associated with overall survival.

Variables	Category	Univariable HR (95% CI)	p-value	Multivariable HR (95% CI)^*^	p-value
Age, year	≤69	1.00 (ref)			
	>69	2.1133 (0.7635–5.8497)	0.1497		
Total white blood cell count (×10^9^/L)	≤9.92	1.00 (ref)			
	>9.92	7.672 (1.7208–34.2056)	0.0075		
Absolute neutrophil count (×10^9^/L)	≤8.95	1.00 (ref)			
	>8.95	15.7741 (2.0613–120.7114)	0.0079		
Lymphocyte count (×10^9^/L)	≤0.81	1.00 (ref)			
	>0.81	0.1047 (0.0137–0.798)	0.0294		
Lactate dehydrogenase (IU/L)	≤356	1.00 (ref)		1.00 (ref)	
	>356	7.7718 (2.1737–27.7873)	0.0016	7.1476 (1.5807–32.3197)	0.0106
CRP (mg/dL)	≤48.8	1.00 (ref)		1.00 (ref)	
	>48.8	7.8242 (2.1874–27.9866)	0.0016	3.7749 (0.8743–16.2995)	0.0751
sCD40 (pg/mL)	≤1804	1.00 (ref)		1.00 (ref)	
	>1804	16.1465 (3.5884–72.6536)	0.0003	6.3586 (1.1813–34.2268)	0.0312
sCTLA-4 (pg/mL)	≤60	1.00 (ref)		1.00 (ref)	
	>60	8.5378 (2.6752–27.2551)	0.0003	5.4644 (1.5099–19.7759)	0.0097
sLAG-3 (pg/mL)	>761	1.00 (ref)		1.00 (ref)	
	≤761	5.7832 (1.9558–17.1004)	0.0015	27.1136 (4.2966–171.0984)	0.0004
sTIM-3 (pg/mL)	≤4752	1.00 (ref)		1.00 (ref)	
	>4752	7.6674 (2.4076–24.4182)	0.0006	5.1311 (1.3950–18.8730)	0.0139
CCL2 (pg/mL)	≤344.26	1.00 (ref)		1.00 (ref)	
	>344.26	21.6145 (2.8206–165.6333)	0.0031	23.6271 (2.5361–220.1196)	0.0055
CCL4 (pg/mL)	≤149.29	1.00 (ref)		1.00 (ref)	
	>149.29	2.969 (1.0117–8.7103)	0.0476	3.4943 (0.6076–20.0972)	0.1610
CXCL10 (pg/mL)	≤157.54	1.00 (ref)		1.00 (ref)	
	>157.54	19.105 (2.4983–146.1022)	0.0045	14.221 (1.6781–120.5176)	0.0149
GM-CSF (pg/mL)	≤61.95	1.00 (ref)		1.00 (ref)	
	>61.95	6.6766 (2.1030–21.1969)	0.0013	5.4952 (1.4981–20.1565)	0.0102
IL-10 (pg/mL)	≤100.01	1.00 (ref)		1.00 (ref)	
	>100.01	6.3218 (2.1954–18.2037)	0.0006	3.9516 (1.1740–13.3004)	0.0265
IL-8 (pg/mL)	≤21.86	1.00 (ref)		1.00 (ref)	
	>21.86	14.8786 (1.9493–133.5644)	0.0092	4.5621 (0.5254–39.6140)	0.1687

^*^Multivariate HR was performed by adjusting for age, sex, absolute neutrophil count, and underlying diseases which may affect prognosis.

HR, hazard ratio; CD, cluster of differentiation; CTLA-4, cytotoxic T-lymphocyte-associated protein 4; LAG-3, lymphocyte-activation gene 3; TIM-3, T-cell immunoglobulin and mucin-domain containing-3; CCL, chemokine CC motif ligand; CXCL, C-X-C motif chemokine ligand; GM-CSF, granulocyte-macrophage colony-stimulating factor; IL, interleukin.

### OS of clusters classified based on K-means analysis

**Figure 3:**
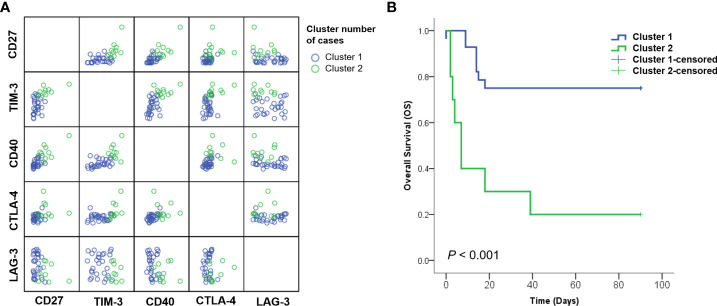
Analysis of patients subdivided into clustering groups. **(A)** Distribution of patients with SARS-CoV-2 classified according to K-means clustering methods. **(B)** Kaplan–Meier curves for overall survival (OS) showed that cluster 2 group with increased sICs, including sCD27, sCD40, sTIM3, and sCTLA-4; cytokines, including CXCL-10 and GM-CSF; and laboratory markers, including CRP and LD exhibited significantly poor prognosis than cluster 1.

Various factors affecting the survival probability of patients with SARS-CoV-2 infection, identified in multivariate analysis, were classified using the K-means clustering method. Upon classification into two clusters, sCD27, sTIM3, sCD40, sCTLA-4, LD, CRP, CXCL10, and GM-CSF were identified as significant clustering factors (**Table 4**, **Figure 3A**). Cluster 2, which included increased levels of sICs and cytokines, along with the laboratory results listed above, was associated with poor prognosis (p< 0.001, **Figure 3B**). In this group, the clustering centers for sCD27, sTIM-3, sCD40, and sCTLA-4 were 2967.4, 5973.2, 3361.1, and 75.3 pg/mL, respectively. The clustering centers of significant cytokines such as CXCL10 and GM-CSF in the cluster 2 group were 637.05 and 102 pg/mL, respectively. In contrast, cluster 1, which showed decreased levels of sICs such as sCD27 (clustering center 818.9 pg/mL), sTIM-3 (3208.2 pg/mL), sCD40 (1558.2 pg/mL), and sCTLA-4 (54.2 pg/mL), and cytokines including CXCL10 (clustering center 272.44 pg/mL) and GM-CSF (55 pg/mL) showed a favorable prognosis (p< 0.001, **Figure 3B**).

**Table 4:** Final cluster centers for variables after K-means analysis and their significance of clustering.

	Final cluster centers		ANOVA					
	Cluster 1	Cluster 2	Cluster		Error		F	p-value
			Mean Square	df	Mean Square	df		
sCD27 (pg/mL)	818.9	2967.4	3.401 × 10^7^	1	1,130,147.134	36	30.095	<0.001
sTIM-3 (pg/mL)	3208.2	5973.2	5.633 × 10^7^	1	1,499,353.034	36	37.570	<0.001
sCD40 (pg/mL)	1558.2	3361.1	2.395 × 10^7^	1	594,688.692	36	40.276	<0.001
sLAG-3 (pg/mL)	1444.5	852.3	2,584,703.687	1	668,155.955	36	3.868	0.057
sCTLA-4 (pg/mL)	54.2	75.3	3287.161	1	254.950	36	12.893	0.001
Age, year	66	73	333.595	1	237.707	36	1.403	0.244
Lactate dehydrogenase (IU/L)	360.75	747.00	1,099,287.829	1	179,843.313	36	6.112	0.018
Absolute neutrophil count (×10^9^/L)	9.99	12.12	33.625	1	29.740	36	1.131	0.295
CRP (mg/dL)	56.83	140.71	51840.489	1	5838.099	36	8.880	0.005
CCL2 (pg/mL)	593.01	693.67	74,655.072	1	664,413.243	36	0.112	0.739
CCL4 (pg/mL)	173	162	987.311	1	15917.210	36	0.062	0.805
CXCL10 (pg/mL)	272.44	637.05	979,509.414	1	211,144.763	36	4.639	0.038
IL-8 (pg/mL)	51.43	41.90	668.424	1	6631.459	36	0.101	0.753
IL-10 (pg/mL)	106	172	32,050.242	1	27,202.981	36	1.178	0.285
GM-CSF (pg/mL)	55	102	15,988.953	1	2860.553	36	5.589	0.024

associated protein 4; LAG-3, lymphocyte-activation gene 3; TIM-3, T-cell immunoglobulin and mucin-domain containing-3; CCL, chemokine CC motif ligand; CXCL, C-X-C motif chemokine ligand; GM-CSF, granulocyte-macrophage colony-stimulating factor; IL, interleukin.

## Discussion

We found that increased sICs, such as sCD40, sCTLA-4, and sTIM-3, and increased cytokines levels, including CCL2, GM-CSF, and IL-10 were significantly poor prognostic factors. These values were significantly correlated with inflammatory markers, such as CRP and procalcitonin, and negatively correlated with the absolute lymphocyte count and platelet count. Cluster analysis of existing laboratory markers with significant sICs and cytokines revealed that patients with higher values of sCD27, sCD40, sCTLA-4, sTIM-3, LD, CRP, CXCL-10, and GM-CSF had a poor prognosis.

We measured the levels of various sICs and cytokines and followed up with each patient at the first and second weeks post-diagnosis. In most previous studies, the days on which sICs or cytokine values were estimated were inaccurate, and the values were measured in the first week of symptom onset (10, 11, 15). Sequential measurement of ICs and cytokine values revealed trends in the changes in IC and cytokine levels and the most favorable measurement time for prognosis prediction. The survivor group showed a significant decrease in the levels of CRP, creatinine and cytokines, such as CXCL10, GM-CSF, and IL-10 in the second week compared with those in the first week. In contrast, in the non-survival group, there was no significant decrease in the levels of laboratory markers, cytokines, or sICs in the first and second weeks. Although no significant differences were observed between the survivor and death groups in the first week, the levels of most markers showed a significant increase in the death group in the second week. Among the laboratory markers, LD, and CRP, and sICs, such as sCD40, and sCTLA-4, and some cytokines (5/11) were significantly higher in the death group than in the survivor group.

ICs play important roles in implanting immune responses that trigger effector functions in various immune cells (3). Although sICs significantly impact viral infection (2, 3, 16), various sICs in patients with SARS-COV-2 infection have not been extensively investigated. Kong et al. recently measured 14 sICs in patients with COVID-19 within three days of hospitalization (10), which showed that sIDO, s4-1BB, sTIM-3, and sCD27 were predictive biomarkers of disease severity. The results showing that sTIM-3 and sCD27 are poor prognostic factors agree with those of the present study. In contrast, we observed an effect on OS by including a large number of deceased patients and by measuring the values at both the first and second weeks; the values measured in the second week were more meaningful than those determined in the first week. Moreover, sCD40 and sCTLA4, which were not included or showed low significance in the above study, were significantly related to OS.

Most studies of sICs in patients with COVID-19 demonstrated the implications of PD-1 or PD-L1 (1, 8, 11, 17). Sabbatino et al. showed that serum PD-L1 levels are prognostic markers in patients with COVID-19 and that PD-L1 dysregulation is associated with COVID-19 pathogenesis (11). However, in our study, the median values of PD-1 and PD-L1 in non-surviving patients were higher than those in surviving patients; however, the difference was not significant. This can be explained by the small sample size of this study. In addition, in a previous study, comparative analysis was performed with healthy controls or non-intensive care unit patients (17), whereas we compared the survival and non-survival of patients with SARS-CoV-2 infection. In another study, the difference between discharged and deceased patients showed a higher p-value than the difference between healthy and discharged patients (11). The low significance of PD-1 and PD-L1 in our study suggests that sICs other than PD-1 and PD-L1 are more useful for predicting the prognosis of SARS-CoV-2-infected patients.

The impact of other sICs has not been widely reported. According to previous studies other sICs, such as CD27, CD40, and T cell exhaustion makers including TIM-3 have been reported to play significant roles in immune-mediated infection control in viral infections. As a member of the TNF superfamily, CD27 includes both costimulatory and apoptosis-inducing molecules, and its stimulation promotes natural killer/T cell survival and effector functions, which are required for the proliferation of various viruses (18–20). In addition, CD40 is important in the IFN-I response, parasitemia control, and host survival (21), and its signaling in macrophages inhibit acute viral replication at the early stages of infection (22). In SARS-CoV-2 infection, CD40 has been used as a subunit vaccine targeting viral antigens to CD40-expressing antigen-presenting cells (23). The newly developed vaccine significantly improved immunity in convalescent macaques, resulting in a reduction in viral load following re-exposure to the virus to levels that may avoid secondary transmission. Finally, as an indicator of CD8+ T cell exhaustion, both PD-1 and TIM-3, CTLA-4, and LAG-3 may be associated with the disease severity of COVID-19. SARS-CoV-2 can lead to limited T-cell function as a result of T-cell exhaustion during long-term infection, which is related to overexpression of immune-inhibitory factors (17, 24). The Gal-9/TIM-3 axis has also been reported to be critical for stratifying patients with SARS-CoV-2 infection with poor prognosis through serial measurements of two patients with COVID-19 (25). In our study, sICs, such as sCD27, sCD40, sCTLA-4, sHVEM, and sTIM-3, but not PD-1 or PD-L1, were significant prognostic factors in patients with SARS-CoV-2 infection. The roles and mechanisms of these sICs in prognosis require further analysis. Particularly, soluble ICs are easy to measure, and serial confirmation of the patient’s status is possible; thus, this approach can be applied in clinical practice.

In this study, various cytokines were measured together with sICs, and clustering analysis was performed by integrating these measured values. Many studies have reported a relationship between the mechanism and prognosis of cytokines in patients with COVID-19 (15, 26–30). However, few studies have focused on the correlation between cytokines and sICs or analyzed them in an integrated manner. In this study, the prognosis-related cytokines, sICs, and clinical laboratory test results were highly correlated. In addition, dividing the patient cluster by integrating various prognostic factors can predict patient mortality. Integrated analysis of various sICs and clinical data can help to accurately determine a patient’s status and auxiliary markers can be used to predict the prognosis of patients with SARS-CoV-2 infection.

One limitation of this study is that only sICs were measured. Further studies are needed to evaluate the levels of IC RNA expression in tissue cultures or tissue biopsy samples *in vitro*. Second, some cytokines could not be analyzed because their levels in patient samples were below the limit of detection. Patients whose serum samples could not be quantified because their cytokines, such as IFN-γ and IL-4, were below the limit of detection were excluded from analysis. This may vary from the results of other studies on cytokines, which is important for prognosis. Third, a comparison with healthy controls was not performed. In a follow-up study, the differences between the SARS-CoV-2-infected group and the healthy control group must be compared and the characteristics of patients with mild symptoms should be analyzed. In addition, during the study period, the vaccination rate in Koreans was 37.4, and 20 out of 38 study participants (52.6%) were sampled after the start date of vaccination against COVID-19. The number of vaccinated patients that can be inferred from this study was less than half and it was difficult to demonstrate the relationship between the status of vaccination and its impact on sICs through this study. Further studies investigating the association with vaccination state would be required.

With the progression of COVID-19, the lack of beds for patients with severe SARS-CoV-2 infection has emerged as an important concern in many countries. It is important to screen patients with COVID-19, predict disease prognosis, identify patients with severe disease, and effectively implement intensive care. Therefore, the markers identified in this study can be used to predict disease severity in patients with SARS-CoV-2 infection. Various sICs such as sCD27, sCD40, sCTLA-4, and sTIM-3 are significant independent prognostic factors and are helpful in prognosis prediction when they are analyzed together with the cytokines and inflammatory markers currently used in clinical practice.

## Data availability statement

The datasets presented in this study can be found in online repositories. The names of the repository/repositories and accession number(s) can be found below: The dataset analyzed in this study can be found in [HARVARD Dataverse] [https://doi.org/10.7910/DVN/TAS2PH].

## Ethics statement

The study was approved by the Institutional Review Board of the Kangnam Sacred Heart Hospital of Hallym University, Seoul, Korea (No. 2020-08-004-003). The ethics committee waived the requirement of written informed consent for participation.

## Author contributions

NL and SJ designed the study; WS supervised the manuscript writing and participated in editing; NL collected the data, performed the statistical analysis, and wrote the manuscript; SJ and KJ participated in manuscript editing; M-JP reviewed the statistical analysis; and all authors reviewed and approved the manuscript.

## Funding

This research was funded by a National Research Foundation of Korea (NRF) grant (grant number NRF-2022R1A2C1003503).

## Acknowledgments

The authors appreciate medical technicians for collecting samples to perform the experiments.

## Conflict of interest

The authors declare that the research was conducted in the absence of any commercial or financial relationships that could be construed as a potential conflict of interest.

## Publisher’s note

All claims expressed in this article are solely those of the authors and do not necessarily represent those of their affiliated organizations, or those of the publisher, the editors and the reviewers. Any product that may be evaluated in this article, or claim that may be made by its manufacturer, is not guaranteed or endorsed by the publisher.
