# Supplementary material for: Prognostic impacts of soluble immune checkpoint regulators and cytokines in patients with SARS-CoV-2 infection
Source: Front Immunol. 2022 Aug 15;13:903419. doi: 10.3389/fimmu.2022.903419 (PMC9423766; doi:10.3389/fimmu.2022.903419)
Supplement: Supplementary file 1 [file Table_1.docx]

**Supplementary Table 1**| Cox proportional hazards model for factors associated with overall survival.

| Variables | Univariable HR (95% CI) | p-value | Multivariable HR (95% CI)^*^ | p-value |
| --- | --- | --- | --- | --- |
| Age (years) | 1.0438 (1.0028–1.0864) | 0.0358 |  |  |
| Total white blood cell count (×10^9^/L) | 1.0997 (1.0121–1.1949) | 0.0248 |  |  |
| Absolute neutrophil count (×10^9^/L) | 1.1124 (1.0240–1.2084) | 0.0117 |  |  |
| Lymphocyte count (×10^9^/L) | 0.1897 (0.0376–0.9573) | 0.0441 |  |  |
| Lactate dehydrogenase (IU/L) | 1.0014 (1.0006–1.0022) | 0.0006 | 1.0019 (1.0009–1.0030) | 0.0002 |
| CRP (mg/dL) | 1.0116 (1.0055–1.0178) | 0.0002 | 1.0175 (1.0082–1.0269) | 0.0002 |
| sCD27 (pg/mL) | 1.0004 (1.0001–1.0007) | 0.0075 | 1.0006 (1.0002–1.0010) | 0.0017 |
| sCD40 (pg/mL) | 1.0009 (1.0005–1.0013) | <0.0001 | 1.0012 (1.0006–1.0017) | 0.0001 |
| sCTLA-4 (pg/mL) | 1.0179 (0.9999–1.0363) | 0.0515 | 1.0308 (1.0069–1.0552) | 0.0113 |
| sLAG-3 (pg/mL) | 0.9992 (0.9986–0.9999) | 0.0289 | 0.9988 (0.9979–0.9996) | 0.0050 |
| sTIM-3 (pg/mL) | 1.0005 (1.0002–1.0009) | 0.0024 | 1.0010 (1.0004–1.0016) | 0.0007 |
| CCL2 (pg/mL) | 1.0005 (1.0001–1.0008) | 0.0091 | 1.0008 (1.0003–1.0013) | 0.0021 |
| CCL4 (pg/mL) | 1.0028 (1.0003–1.0054) | 0.0314 | 1.0029 (0.9995–1.0062) | 0.0933 |
| CXCL10 (pg/mL) | 1.001 (1.0003–1.0017) | 0.0047 | 1.0006 (0.9998–1.0014) | 0.1415 |
| GM-CSF (pg/mL) | 1.0109 (1.0036–1.0182) | 0.0035 | 1.0095 (1.0014–1.0176) | 0.0213 |
| IL-10 (pg/mL) | 1.003 (1.0010–1.0050) | 0.003 | 1.0042 (1.0016–1.0069) | 0.0018 |
| IL-8 (pg/mL) | 1.0049 (1.0012–1.0087) | 0.01 | 1.0053 (1.0006–1.0101) | 0.0279 |

^*^ Multivariate HR was performed by adjusting for age, sex, absolute neutrophil count, and underlying diseases which may affect prognosis.

Abbreviations: HR, hazard ratio; CD, cluster of differentiation; CTLA-4, cytotoxic T-lymphocyte-associated protein 4; LAG-3, lymphocyte-activation gene 3; TIM-3, T-cell immunoglobulin and mucin-domain containing-3; CCL, chemokine CC motif ligand; CXCL, C-X-C motif chemokine ligand; GM-CSF, granulocyte-macrophage colony-stimulating factor; IL, interleukin
